# Neurologic Manifestations Associated with an Outbreak of Typhoid Fever, Malawi - Mozambique, 2009: An Epidemiologic Investigation

**DOI:** 10.1371/journal.pone.0046099

**Published:** 2012-12-03

**Authors:** James Sejvar, Emily Lutterloh, Jeremias Naiene, Andrew Likaka, Robert Manda, Benjamin Nygren, Stephan Monroe, Tadala Khaila, Sara A. Lowther, Linda Capewell, Kashmira Date, David Townes, Yanique Redwood, Joshua Schier, Beth Tippett Barr, Austin Demby, Macpherson Mallewa, Sam Kampondeni, Ben Blount, Michael Humphrys, Deborah Talkington, Gregory L. Armstrong, Eric Mintz

**Affiliations:** 1 Division of High-Consequence Pathogens and Pathology, National Center for Emerging and Zoonotic Infectious Diseases (NCEZID), Centers for Disease Control and Prevention (CDC), Atlanta, Georgia, United States of America; 2 Scientific Education and Professional Development Program Office, Epidemic Intelligence Service, CDC, Atlanta, Georgia, United States of America; 3 Current Position, New York State Department of Health, Albany, New York, United States of America; 4 Ministry of Health, Maputo, Mozambique; 5 Ministry of Health, Lilongwe, Malawi; 6 Division of Foodborne, Waterborne, and Environmental Infectious Diseases, NCEZID, CDC, Atlanta, Georgia, United States of America; 7 Division of Environmental Hazards and Health Effects, National Center for Environmental Health (NCEH), CDC, Atlanta, Georgia, United States of America; 8 Global AIDS Program, CDC, Lilongwe, Malawi; 9 Malawi-Liverpool-Wellcome Trust, College of Medicine, Blantyre, Malawi; 10 Division of Viral Diseases, National Center for Immunization and Respiratory Diseases, CDC, Atlanta, Georgia, United States of America; The Australian National University, Australia

## Abstract

**Background:**

The bacterium *Salmonella enterica* serovar Typhi causes typhoid fever, which is typically associated with fever and abdominal pain. An outbreak of typhoid fever in Malawi-Mozambique in 2009 was notable for a high proportion of neurologic illness.

**Objective:**

Describe neurologic features complicating typhoid fever during an outbreak in Malawi-Mozambique

**Methods:**

Persons meeting a clinical case definition were identified through surveillance, with laboratory confirmation of typhoid by antibody testing or blood/stool culture. We gathered demographic and clinical information, examined patients, and evaluated a subset of patients 11 months after onset. A sample of persons with and without neurologic signs was tested for vitamin B6 and B12 levels and urinary thiocyanate.

**Results:**

Between March – November 2009, 303 cases of typhoid fever were identified. Forty (13%) persons had objective neurologic findings, including 14 confirmed by culture/serology; 27 (68%) were hospitalized, and 5 (13%) died. Seventeen (43%) had a constellation of upper motor neuron findings, including hyperreflexia, spasticity, or sustained ankle clonus. Other neurologic features included ataxia (22, 55%), parkinsonism (8, 20%), and tremors (4, 10%). Brain MRI of 3 (ages 5, 7, and 18 years) demonstrated cerebral atrophy but no other abnormalities. Of 13 patients re-evaluated 11 months later, 11 recovered completely, and 2 had persistent hyperreflexia and ataxia. Vitamin B6 levels were markedly low in typhoid fever patients both with and without neurologic signs.

**Conclusions:**

Neurologic signs may complicate typhoid fever, and the diagnosis should be considered in persons with acute febrile neurologic illness in endemic areas.

## Introduction

Typhoid fever is a bacterial disease caused by infection with *Salmonella enterica* serovar Typhi (*Salmonella* Typhi). It is transmitted through the fecal-oral route, generally by contaminated water or food. Typically, it presents as an acute febrile illness often accompanied by signs and symptoms such as headache, abdominal pain, diarrhea or constipation, and malaise [1]. Other, more severe complications of typhoid fever include intestinal perforation, hepatitis, pneumonia, and tissue abscesses [1], [2]. Neurologic illness has also been described, most frequently as acute encephalopathy or meningitis [3]. A variety of objective neurologic signs have been documented, including acute neuropsychiatric illness [4], [5], [6], spasticity and clonus [4], [7], ataxia [8], [9], [10], [11], [12], [13], aphasia [14], [15], [16], and cerebritis [3], [17]. However, these findings have generally appeared as case reports or small case series.

Beginning in June 2009, an outbreak of unexplained febrile illness occurred in villages along the border region between southern Malawi and western Mozambique. This area was known to have a high rate of general mild malnutrition, with most diets high in consumption of wheat, corn, and leafy vegetables. Cassava is consumed, but infrequently. Initial reports described many persons who presented with acute neurologic illness including mental status changes, headache, “difficulty walking”, dysarthria, and hyperreflexia. Other neurologic features including seizures and neck stiffness were also described. Gastrointestinal complaints were not prominent among patients early in the outbreak. The investigators initially suspected common etiologies of such neurologic abnormalities in sub-Saharan Africa such as acute encephalitis or heavy metal toxicity, as well as less common etiologies such as neurolathyrism and konzo. However, subsequent investigation revealed the outbreak to be caused by typhoid fever, and after the etiology was determined, persons with signs and symptoms more typical of typhoid fever were increasingly recognized.

We describe the results of an investigation into the clinical, neurologic and laboratory features of persons with typhoid fever during this outbreak. Our investigation suggests that signs of upper motor neuron dysfunction were predominant, neurologic features were generally a later manifestation of typhoid fever, and outcome was generally favorable.

## Methods

### Patient Identification

The outbreak was first noted in June 2009 by health personnel in Neno District, Malawi, who observed an increase in patients hospitalized at Neno District Hospital with fever and neurologic illness. Ill patients were from villages in Neno District and neighboring Tsangano District, Mozambique. The outbreak occurred in a remote location; the closest health center, Nsambe Health Centre, is approximately 8.5 km away by dirt road over rough terrain. As cases continued, a larger investigation was initiated by the Malawi Ministry of Health (MOH).

**Figure 1:**
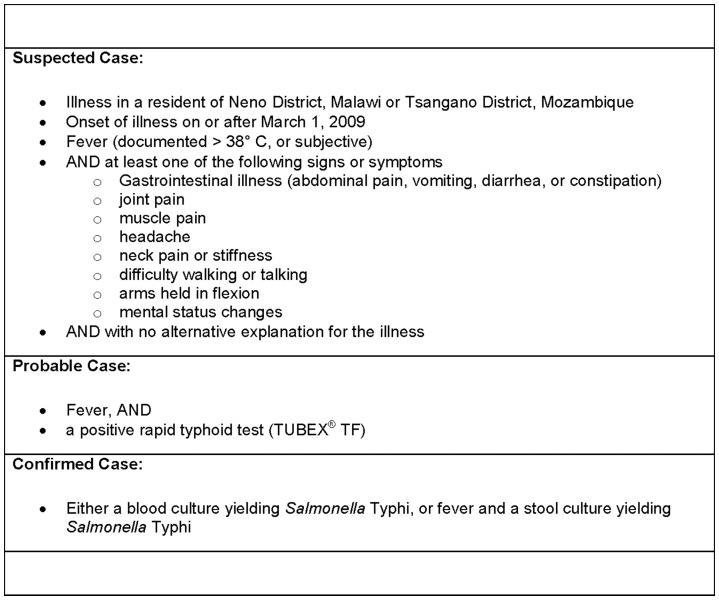
Case definition for typhoid fever used in outbreak investigation, Malawi/Mozambique.

Between July and November, 2009, an epidemiologic investigation was conducted [18] that included structured retrospective interviews of previously ill individuals to determine initial signs and symptoms, risk factors, and possible exposures; retrospective hospital record review; structured interview and clinical examination of acutely ill individuals; and verbal autopsy for deceased patients. Questions included specific assessment of dietary habits, sources of drinking water, and other possible exposures that might lead to an infectious or toxic etiology or result in a nutritional deficiency, Based upon initial clinical findings and laboratory results among outbreak patients, a case definition was established for suspected, probable, and confirmed cases [18] (Figure 1). Active surveillance was implemented in affected villages to identify possible patients presenting early in the course of illness.

We identified patients with neurologic illness by several methods. We reviewed medical records at Neno District Hospital for admissions during March through November 2009 for descriptions of objective neurologic findings. From July 22 through November 13, 2009, we prospectively gathered data on persons presenting with illness, and hospitalized patients meeting the case definition underwent neurologic evaluation by one of the authors, a U.S. board-certified neurologist (JJS). When possible, suspected patients meeting clinical case criteria but not hospitalized were evaluated in their villages. When possible, hospitalized patients were serially re-evaluated in order to document progression of illness; a subset of patients underwent re-evaluation approximately 11 months after acute illness to detect the presence of long-term neurologic sequelae.

### Laboratory Testing

Cerebrospinal fluid (CSF) white blood cell count (WBC), glucose, and protein level results were reviewed when available. Clinical specimens obtained from acutely ill patients, including serum, blood, urine, and CSF, initially underwent testing for a number of infectious agents including various viruses, bacteria, parasites, and rickettsiae, as well as various toxins at laboratories at CDC (Table S1). Typhoid-specific testing in the field was performed using a rapid diagnostic assay (TUBEX® TF, IDL Biotech, Bromma, Sweden) that detects IgM antibodies against the O9 lipopolysaccharide antigen of *Salmonella* Typhi. Following the establishment of field capacity to collect and transport specimens for culture, confirmatory testing for typhoid was performed by blood or stool culture.

Because some of the neurologic features observed in the patients were similar to those seen in some micronutrient abnormalities, we tested serum specimens on a subset of patients with and without neurologic illness for vitamin B6 and B12 concentrations. Serum B12 concentrations were assessed by electrochemiluminescence immunoassay (ECLIA; Roche Diagnostics Modular Analytics E170) at CDC. Serum B6 concentrations [pyridoxal 5′-phosphate (PLP) and 4-pyridoxic acid (4PA)] were assessed by high-performance liquid chromatography with fluorometric detection [19]. PLP is indicative of longer-term vitamin B6 status, while 4PA is indicative of short-term vitamin B6 intake. Since the neurologic presentation including spasticity and clonus appeared similar to konzo, an illness due to cyanogen toxicity from consumption of improperly cooked bitter cassava, we also tested urine specimens from a subset of patients with and without neurologic illness for urinary thiocyanate levels, a marker for cyanogenic compound exposure. Urine thiocyanate was quantified using isotope dilution tandem mass spectrometry at CDC [20].

### Neurodiagnostic Testing

Three patients with neurologic illness underwent brain and spinal cord magnetic resonance imaging (MRI) at the Malawi MRI Facility in Blantyre. Autopsy was performed on one decedent with neurologic illness; tissue from central nervous system, meninges, lung, spleen, kidney, and liver was assessed by routine histology at University of Malawi Medical School, and immunohistochemical staining for leptospira and flaviviruses at CDC.

### Data Analysis

Data were entered into an Access™ database. Mean and median concentrations of vitamins B6 and B12 and urinary thiocyanate were calculated; comparisons between patients with and without neurologic illness were made using Wilcoxon rank-sum analysis. SAS software version 9.2 (SAS Institute Inc, Cary, NC) was used for analyses.

### Determination of Possible Subclinical Community Neurologic Findings

To determine the possible presence of subclinical or mildly clinical upper motor neuron findings among village populations, persons in two affected villages, who were approximately representative of age group and sex distribution of typhoid cases were evaluated for the presence of objective neurologic findings during two village-wide evaluations. These persons underwent a screening neurological evaluation by one of the authors (JJS).

The Malawi MOH conducted the investigation in the context of an outbreak response and a public health intervention, and it was determined by human subjects review at CDC to be public health response evaluation and not research. Verbal consent was obtained from patients or guardians for collection of biological specimens and physical examination.

## Results

### Demographics

Between March 1 and November 13, 2009, we identified 303 persons meeting the case definition for typhoid fever, including 212 suspected, 45 probable, and 46 confirmed cases. Of these, 40 (13%) persons had objective, focal neurologic findings documented in the medical chart (n = 6) or elicited on examination (n = 34); an additional 27 persons had encephalopathy or altered mental status but did not demonstrate focal neurologic findings and were not included in subsequent analysis. Twenty-six of the 40 cases with neurologic signs met criteria for a suspected case, 10 for a probable case, and 4 for a confirmed case. The median age was 18 years (range: 3–57 years); 53% were female. Age and sex distribution were not significantly different between patients with and without neurologic illness (data not shown). Twenty-seven persons (68%) with neurologic illness were hospitalized, and there were five (13%) deaths; one decedent underwent autopsy. Twenty-one (53%) persons were treated with a variety of antimicrobials at some point during their illness, generally upon hospital admission. The most commonly administered antimicrobials included chloramphenicol (n = 15), lumefantrine-artemether (n = 9), and penicillin G (n = 7). Specific information on dose and duration of antimicrobial therapy was not available.

### Clinical Findings

Neurologic signs among cases are shown in Table 1. Ascertainment of exact dates of onset of illness was difficult in this population; however, the mean interval between the best estimate of onset of illness and first documentation of neurologic signs (generally obtained at time of presentation) was 12.4 days (range, 1–35 days). The most common neurologic manifestations were upper motor neuron signs including deep tendon hyperreflexia, spasticity, and sustained ankle clonus; 17 patients had a constellation of all three of these signs. The lower extremities were most frequently affected, and spasticity frequently resulted in gait disturbance. Babinski's signs were present in five patients with other upper motor neuron features; bowel or bladder dysfunction was generally absent. Significant sensory abnormalities, including decrease of vibratory and position sense, were not observed. Twenty patients demonstrated truncal or appendicular ataxia or both, which also resulted in gait abnormalities. Ataxia was unaccompanied by cerebellar signs such as nystagmus or intention tremor. Eight patients demonstrated moderate to severe features of parkinsonism, including bradykinesia, postural instability, facial masking, and decreased arm swing. Other commonly observed neurologic features included flaccid dysarthria with hypophonetic speech (n = 21) and static/kinetic tremor (n = 4). In addition to neurologic signs, many patients described subjective neurologic symptoms including hearing loss and visual problems.

**Table 1:** Neurologic signs and symptoms among 40 persons with neurologic illness associated with typhoid fever.

Neurologic Sign/Symptom	N	%
Upper Motor Neuron Signs		
Hyperreflexia	22	55
Sustained ankle clonus	16	40
Spasticity	10	25
Babinski's sign	5	13
Dysarthria	21	53
Ataxia	22	55
Encephalopathy/altered mental status	15	38
Headache	15	38
Hearing loss (subjective)	9	23
Parkinsonism	8	20
Tremor	4	10

Eleven patients had CSF examination; WBC, protein, and glucose levels were within normal limits in all. No routine blood laboratory parameters were consistently abnormal among patients. Extensive diagnostic testing for other viral, bacterial, parasitic, and rickettsial pathogens, including broad-spectrum polymerase-chain reaction (PCR) testing and random-primer sequencing, was performed on 16 of the 303 patients overall and included four patients with neurologic illness. Viral cultures, serologic assays for infectious agents, and PCR for pathogen-specific nucleic acid sequences were negative, and random-primer PCR assays in serum were unremarkable or nonspecific. Autopsy specimens from one decedent with focal neurologic findings, including ataxia, spasticity, and clonus, showed patchy necrosis in the liver; histopathology of cerebral cortex, cerebellum, pons, and medulla were unremarkable and without perivascular cuffing or other signs of acute inflammation. Immunohistochemical assays for leptospira and flaviviruses in all tissues were negative.

### Dietary Findings and Laboratory Results

Although chronic malnutrition was present in this poor and rural area, there had been no acute changes in food availability or food type consumption reported by villagers. Cassava consumption was reported in all affected areas, including both bitter and sweet cultivars, but no recent changes in cassava processing were reported. We were unable to elicit a history of pea or legume consumption, or other plants that would be suggestive of *Lathyrus sativus*. Serum vitamin B12 concentrations were assessed in 13 patients with and 10 patients without neurologic signs. The distribution of the 23 levels (range: 175–1540 pg/mL) was mainly within the central 95 percent reference interval generated from a sample of presumably healthy U.S. residents, and the distributions of the two groups were not significantly different from each other (Table 2). Only one patient (without neurologic signs) had a serum vitamin B12 concentration <200 pg/mL, a cutoff value often used to indicate B12 deficiency [21]. Serum PLP and 4-PA concentrations were assessed in eight persons with and nine without neurologic illness. The distribution of these 17 PLP concentrations (range: 0.7–60.4 nmol/L) was lower than the central 95 percent reference interval generated from a U.S. population [22], while the 4PA concentrations (range: 7.4–456 nmo/L) were mainly within this referent range. Fourteen (82%) of the samples had low PLP values (<20 nmol/L) indicative of B6 deficiency, a higher percentage than that seen in the U.S. population (25% of non-supplement users) [22]. The PLP and 4PA concentrations in the two groups were characterized by a large variance, making it difficult to assess statistical differences. However, the group with neurologic signs did not appear to have lower PLP and 4PA values than the group without neurologic signs. Urinary thiocyanate levels among 16 persons without neurologic illness were significantly higher than in 5 persons with neurologic illness (p = 0.004, Table 2); however, concentrations in all but one (without neurologic signs) were within the established reference range [23], and no urinary thiocyanate concentrations were above those previously associated with health effects [24].

**Table 2:** Levels of serum vitamin B12, vitamin B6 (PLP and 4PA), and urine thiocyanate In typhoid fever patients with and without neurologic signs.

	*Neurologic Signs*			*No Neurologic Signs*			
Assay	N	Median	Mean (95% CI)	N	Median	Mean (95% CI)	Referent Range¥
**Serum Vitamin B12 (pg/ml)**	13	400	597 (367–828)	10	377	415 (280–550)	211–946¥
**Serum Vitamin B6 (PLP** # **) [nmol/L]**	8	2.1	12.6 (0–30.8)£	9	3.2	6.5 (0.6–12.3)	11.0–337∧
**Serum Vitamin B6 (4PA** * **) [nmol/L]**	8	20.0	72.6 (0–202.2)£	9	12.5	26.1 (1.2–51.0)	8.8–464∧
**Urine Thiocyanate (ng/ml)**	5	112.5	209.6 (0–446.2)	16	1,185.0	1,407.0 (806.7–2008.5)	1,000–4,000€

¥ Referent ranges for vitamin B12 obtained from kit manufacturer, based upon presumably healthy US population [1].

∧ Referent ranges for vitamin B6 (PLP and 4PA) obtained from a subset of samples from US National Health and Nutrition Examination Survey (NHANES) data among a presumably healthy US population [2].

€ Referent ranges for urine thiocyanate levels obtained from a sample of non-smoking US residents [3].

# PLP – Pyridoxal 5′ phosphate.

* 4PA – 4-pyridoxic acid.

£ Calculated lower confidence interval limits for PLP and 4PA resulted in negative values; for the purposes of reporting, a lower limit of 0 was used as the lower 95% confidence interval limit.

### Neuroimaging Findings

**Figure 2:**
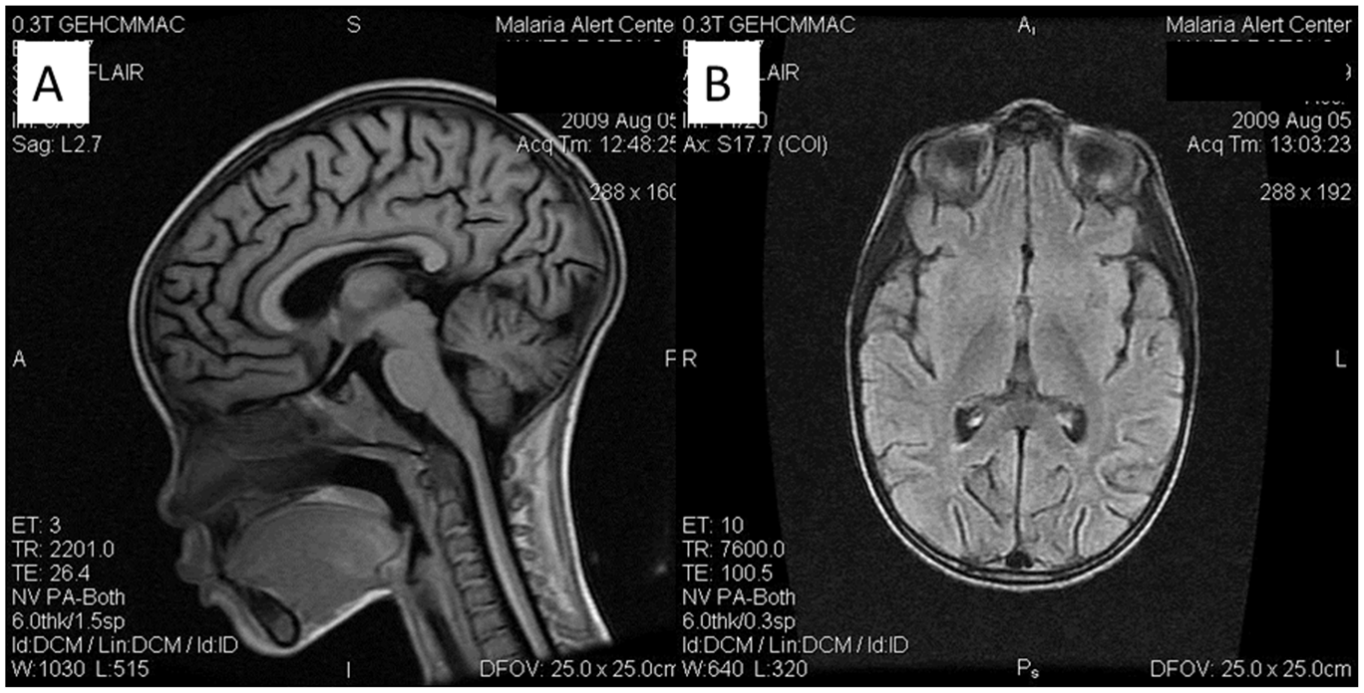
Magnetic resonance imaging (MRI) of a patient with neurologic illness associated with typhoid fever, Malawi. Coronal T1 FLAIR (A) and axial T2 FLAIR (B) MRI Images demonstrating generalized cerebral atrophy, 7 year-old male with neurologic illness associated with acute typhoid fever, Malawi.

MRI of the brain and spinal cord was performed on three symptomatic persons (two probable, one suspect) with neurologic signs including spasticity, ataxia, and parkinsonism, during their acute illnesses. No signal abnormalities were present, however all three demonstrated generalized cerebral atrophy disproportionate to age (5, 7, and 18 years) (Figure 2).

### Short-Term Outcomes

Seventeen typhoid fever patients hospitalized with neurologic illness were serially evaluated for at least 1 week following onset. All had upper motor neuron signs in combination with other neurologic features; seven had parkinsonism. By 1 month after onset, seven had normal neurologic examinations and were symptomatically well with complete resolution of neurologic signs. By 2 months, an additional 6 had resolution of neurologic signs (Table 3). Four continued to display objective neurologic findings—an 18-year-old male with persistent myoclonus, ataxia, and spasticity; a 14-year-old female with persistent ataxia and parkinsonism; and a 7-year-old male and 16-year-old female both of whom had persistent lower extremity hyperreflexia, clonus, and spastic gait. Thirteen of these patients were re-assessed at approximately 11 months after acute illness; none had experienced a recurrence of neurologic illness, but the two patients with hyperreflexia, clonus, and spasticity at one month continued to demonstrate these signs.

**Table 3:** Demographics, clinical features, and outcomes of 17 persons with typhoid-associated neurologic illness, Malawi-Mozambique, 2009.

Age	Sex	Initial Signs/Symptoms	Interval, Illness Onset to Neurologic Signs (Approx.)	Neurologic features	Follow-Up	Additional Studies/Comment
5	M	Fever, abdominal pain	5	Hyperreflexia, sustained ankle clonus, diffuse kinetic tremors, severe ataxia	Normal exam at 2 and 11 month evaluations	CSF: WBC 0 cells/mm^3^, protein 16 mg/dL, glucose 64 mg/dLMRI: moderate generalized cerebral atrophy
7	M	Fever, headache	12	Hyperreflexia, lower extremity spasticity, sustained ankle clonus, truncal ataxia	Normal exam at 2 and 11 month evaluations	MRI: mild generalized cerebral atrophy
7	M	Fever, abdominal pain, back pain	NK	Lower extremity hyperreflexia, sustained ankle clonus, mild truncal ataxia	Persistent lower extremity hyperreflexia, clonus, spasticity at 1 month; unchanged at 11 months	
10	M		NK	Hyperreflexia, lower extremity spasticity, sustained ankle clonus, parkinsonism	Normal exam at 2 and 11 month evaluations	
13	M	Fever, abdominal pain, backache, leucopenia	28	Lower extremity hyperreflexia, sustained ankle clonus, parkinsonism	Normal exam at 2 and 11 month evaluations	
13	M	Fever, myalgias, back and neck pain, “difficulty walking”	NK	Lower extremity hyperreflexia, sustained ankle clonus, left Babinski's sign	Normal exam at 1 and 11 month evaluations	
14	F	Fever, leg pain	3	Diffuse hyperreflexia, truncal and appendicular ataxia,parkinsonism	Persistent ataxia and parkinsonism at 1 month	
14	M	Fever, myalgias, cough, leg pain	4	Lower extremity hyperreflexia, sustained ankle clonus, truncal ataxia, parkinsonism	Normal exam at 1 and 11 month evaluations	CSF: WBC ND, protein 21 mg/dL, glucose 66 mg/dL
16	F	Fever, abdominal pain, leg pain	13	Lower extremity hyperreflexia, sustained ankle clonus, spasticity, diffuse myoclonus, orthostatic tremor	Persistent lower extremity hyperreflexia, clonus, spasticity at 1 month; unchanged at 11 months	
17	F	Fever, neck and back pain, loose stools	14	Lower extremity hyperreflexia, sustained ankle clonus, altered mental status/encephalopathy	Normal exam at 2 and 11 month evaluations	
18	F	Fever, headache, abdominal pain, dizziness	21	Lower extremity hyperreflexia, sustained ankle clonus,parkinsonism, myoclonus	Normal exam at 2 and 11 month evaluations	
18	F	Headache	7	Hyperreflexia, sustained ankle clonus, truncal and appendicular ataxia, parkinsonism, confusion/altered mental status, ataxic dysarthria, diffuse kinetic tremors	Normal exam at 2 and 11 month evaluations	CSF: WBC 1 cell/mm^3^, protein 20 mg/dL, glucose 52 mg/dLMRI: moderate generalized cerebral and cerebellar atrophy
18	M	Fever, myalgias, back pain, headache, “difficulty walking”	NK	Lower extremity hyperreflexia, lower extremity spasticity, diffuse myoclonus, subjective hearing loss	At 1 month evaluation: clinical status unchanged	
19	M	Fever, backache	35	Hyperreflexia, sustained ankle clonus, truncal ataxia, diffuse kinetic tremors	Normal exam at 2 and 11 month evaluations	
26	F	Fever	30	Hyperreflexia, sustained ankle clonus, truncal and appendicular ataxia,mild parkinsonism	Normal exam at 2 and 11 month evaluations	
52	F	Fever, headache, myalgias, abdominal pain, joint pain	3	Lower extremity hyperreflexia, sustained ankle clonus, lower extremity spasticity, truncal ataxia; subjective hearing loss	Normal exam at 1 month evaluation	
57	F	Fever, chills, general body pain, “difficulty walking”	21	Lower extremity hyperreflexia, sustained ankle clonus; severe hip flexor and extensor weakness	Normal exam at 1 month evaluation	

CSF: Cerebrospinal fluid.

WBC: White blood cell count.

NK: Not known.

### Assessment of Subclinical Neurological Illness Among Unaffected Villages

Sixty-five persons from two affected villages who were without a history of illness compatible with typhoid within the previous 6 months, and did not describe a prior history of possible neurologic illness, underwent screening neurologic examination, 35 from Dackson, Mozambique, and 30 from Nseula, Malawi. Median age of these persons was 16 years (range, 4–78 years), and 54% were female, which was similar to the age and sex distribution of patients with neurologic illness (data not shown). Three (5%) persons (median age, 19 years) had brisk deep tendon reflexes with crossed adductors; 1 of these had sustained (>5 beats) ankle clonus. No history of prior neurologic illness could be elicited from these persons. Other nonspecific findings, including physiologic tremors and lower extremity areflexia, were observed in 9 persons.

## Discussion

This outbreak of typhoid fever in an area along the Malawi- Mozambique border was associated with a range of objective neurologic findings. Although neurologic complications of typhoid fever have been previously described, the prominence of neurologic illness early in the outbreak initially led to diagnostic confusion and caused investigators to consider numerous other etiologies thought more likely to result in acute febrile neurologic illness. Our investigation benefitted from detailed clinical information, extensive testing for other possible etiologies of neurologic illness, and laboratory confirmation of a large number of temporally and spatially clustered cases.

Thirteen percent of the 303 persons meeting case definition criteria for typhoid fever in this outbreak demonstrated objective neurologic illness. Neurologic signs have been previously described in association with typhoid fever, and have commonly included spasticity and clonus, ataxia, and dysarthria, and less frequently, neuropsychiatric features [5], [25], [26], cerebellar dysfunction [17], and ophthalmoplegia or other cranial nerve abnormalities [27], [28], [29]. However, most descriptions of neurologic complications of typhoid fever have been from case reports or small case series, and laboratory confirmation of acute typhoid fever is often absent. To our knowledge, this is the first description of prominent neurologic findings associated with typhoid fever in an outbreak setting. Because outbreaks often result in persons with similar environmental exposures, and, in some cases, genetic factors, being affected, the relatively large number of persons presenting with neurologic illness in this setting may be of etiologic significance.

The most common manifestations in our patients were related to upper motor neuron dysfunction, including spasticity, clonus, and hyperreflexia; a bradykinetic–rigid syndrome; and ataxia. Other manifestations, including seizures, tremors, and dysarthria, were also observed. The presence of variable neurologic manifestations suggests that typhoid produces dysfunction at numerous sites within the nervous system. Many patients presented with neurologic findings in the absence of encephalopathy or other alteration in mental status, indicating that typhoid may produce focal, as well as generalized, neurologic dysfunction. With few exceptions, the neurologic findings in these subjects resolved over time, sometimes within weeks of acute illness, and long-term or recurrent neurologic sequelae were largely absent among a subset of persons we were able to assess in extended follow-up. Notably, we did not observe some of the other neurologic manifestations that have been frequently mentioned in the setting of typhoid fever, such as acute psychosis [6], [25], acute inflammatory polyradiculoneuropathy [15], [30], or focal cortical signs [14], [15], [16].

The reason for the high proportion of cases with neurologic illness during this outbreak is unclear, but there are several possibilities. Surveillance bias is possible; early surveillance and case detection efforts focused on those persons hospitalized with neurologic features. Following recognition of typhoid as the cause of the outbreak, more persons with features typical of typhoid fever, including abdominal pain and other gastrointestinal symptoms, were detected. The involvement of neurologists in the outbreak investigation possibly led to detection of neurologic features that might not be typically assessed or noted by other clinicians. Neurologic manifestations of typhoid have been described as a late manifestation of illness [5], [31], [32], and the median interval between symptom onset and documentation of neurologic signs in our patients was 12 days. Several factors, including delayed presentation to clinical care and ineffective antimicrobial treatment early in the outbreak because of multi-drug resistance of the causative *Salmonella* Typhi strain [18] may have led to a prolonged course of illness early in the outbreak, resulting in a greater prevalence of neurologic signs. Importantly, following implementation of early diagnostic capabilities and appropriate definitive antimicrobial treatment of typhoid fever with ciprofloxacin, the number of persons presenting with neurologic illness appeared to decrease, suggesting that prompt treatment may avert the onset of neurologic illness.

The mechanism by which typhoid fever may produce neurologic illness is unknown. Rare cases of *Salmonella* Typhi bacterial meningitis, meningo-encephalitis, and intracranial abscesses have been reported both in children and adults [3], [33], [34]. However, a neuroinvasive bacterial process appears unlikely in our patients; CSF was generally unremarkable and without pleocytosis or protein elevation, features of meningismus were generally absent, and CNS tissue from one confirmed case with neurologic illness, as well as brain and spinal cord MRI on 3 acutely ill patients, did not demonstrate signs of inflammation. This is consistent with prior reports in which neurologic manifestations have largely been unassociated with evidence of CNS inflammation. For these same reasons, a para- or post-infectious immune-mediated inflammatory process in the majority of cases would seem unlikely.

An underlying host factor or environmental exposure that may predispose persons to develop neurologic illness in the setting of severe systemic infection due to typhoid is possible. Many of the predominant signs and symptoms observed in these patients, including spasticity, clonus, hyperreflexia, and ataxia, may be seen with micronutrient abnormalities including vitamin B6 toxicity/deficiency and B12 deficiency [35], [36], [37], [38]. We assessed these micronutrient levels in a subset of persons with and without neurologic signs and did not detect significant differences; however, our sample size was small and variability in the data made it difficult to assess statistical differences. The upper motor neuron findings in this population initially appeared similar to konzo, a neurologic illness seen in tropical areas and associated with thiocyanate toxicity due to consumption of inadequately cooked bitter cassava [39], [40], [41]. The ataxia demonstrated by some of our patients resembled tropical ataxic neuropathy, also related to the dietary use of large quantities of cassava over long periods of time [42], [43]. Although we considered these etiologies because cassava was part of the local diet, the often dramatic and complete resolution of neurologic signs in our patients is inconsistent with konzo or tropical ataxic neuropathy, and measurement of urinary thiocyanate levels did not demonstrate evidence of acute or chronic cyanogen toxicity. Similarly, neurolathyrism seemed unlikely due to improvement in neurologic signs, and we could not obtain a history of consumption of peas or legumes [44], [45].

Production of a bacterial toxin may lead to neurologic illness, with toxins produced by *Clostridium botulinum* and *Corynebacterium diphtheriae* being fundamental examples [46]. The diffuse nature of neurologic involvement observed with typhoid-associated disease, and the apparent reversibility of these signs may be suggestive of a bacterial toxic etiology. *Salmonella* Typhi produces a cytolethal toxin, but the role of this toxin in the pathogenesis of typhoid fever is unknown [47], [48]. Isolates of *Salmonella* Typhi obtained from cases in this outbreak, including persons with neurologic illness, did not demonstrate significant differences in genetic or bacteriologic properties from other isolates in central Africa [18]; further investigations into the possible presence of a *Salmonella* Typhi-produced neurotoxin are ongoing. Some viral and bacterial infections have been proposed to result in a “cytokinemia” in which hyper-reactive pro- and anti-inflammatory cytokines result in alterations of CNS function with encephalopathy and neurologic illness [49], [50], [51]. While our investigation did not include assessments of cytokine function, such an indirect effect of *Salmonella* Typhi on the nervous system should be explored in future studies.

Our study has limitations. We did not perform neurologic examinations on all outbreak patients, and the number of cases of neurologic illness may have been underestimated or otherwise biased. Not all patients with suspected illness were positive for or underwent testing for *Salmonella* Typhi infection, and misclassification of some cases is possible. While we initially screened a large number of cases for numerous other infectious and toxic etiologies of neurologic illness, other alternative or concomitant causes of febrile neurologic illness in these persons cannot be entirely excluded. HIV, which could certainly be a contributory factor in neurologic illness occurring in this population, was not routinely tested for. Baseline neurologic status on these patients was unknown, and some may have demonstrated neurologic findings unrelated to their typhoid illness. Specifically, the presence of disproportionate cerebral atrophy on MRI in 3 cases suggests that other host factors, such as nutritional deficiencies, prior cerebral infections, antenatal/perinatal insults, or other factors may result in a background level of mild neurologic illness in this population. It is possible that severe systemic illness caused by typhoid fever, or a *Salmonella* Typhi-specific factor, may exacerbate mild or subclinical neurologic deficits.

Our study demonstrates that persons with typhoid fever may develop acute and severe neurologic illness. The underlying pathophysiological mechanisms producing these features remain unknown. The varying neurologic manifestations observed in this group of patients with typhoid-associated neurologic illness suggest involvement of multiple nervous system localizations. Neurologic illness associated with typhoid fever appears to resolve over time, with few ongoing sequelae, a feature that is important in the prognostic assessment of cases. Acute infection with *Salmonella* Typhi should be included in the differential diagnosis of persons originating or traveling from a typhoid-endemic area with acute febrile neurologic illness, particularly if viral and bacterial etiologies more typically associated with neurologic illness are not apparent. A better understanding of the underlying pathophysiologic mechanisms associated with neurologic illness in typhoid fever is needed.

## Supporting Information

Table S1 **Initial pathogen testing among ill persons during outbreak of typhoid fever, Malawi – Mozambique, 2009**
(DOC)

Video S1 **18-year old female with typhoid-associated neurologic illness demonstrating severe truncal and appendicular ataxia and facial masking.**
(WMV)
