# Supplementary material for: Neurologic Manifestations Associated with an Outbreak of Typhoid Fever, Malawi - Mozambique, 2009: An Epidemiologic Investigation
Source: PLoS One. 2012 Dec 3;7(12):e46099. doi: 10.1371/journal.pone.0046099 (PMC3513310; doi:10.1371/journal.pone.0046099)
Supplement: Table S1 — Initial pathogen testing among ill persons during outbreak of typhoid fever, Malawi – Mozambique, 2009 (DOC) [file pone.0046099.s001.doc]

| **Table S1.** | |
| --- | --- |
| Serum Polymerase Chain Reaction (PCR) | Serum Random-Primer PCR Sequencing |
| - *Enteroviruses* | Serum 16S Ribosomal PCR |
| - *Cardioviruses* | Serologic Testing |
| - *Parechoviruses* | - *Yellow fever virus* |
| - *Alphaviruses* | - *West Nile virus* |
| - *Adenoviruses* | - *Chikungunya virus* |
| - *Bornaviruses* | - *Dengue virus* |
| - *Bunyaviruses* | - *Tahy na virus* |
| - *Coronaviruses* | - *HTLV-1* |
| - *Flaviviruses* | - *Simian foamy virus* |
| - *Herpesviruses* | Malaria smears, malaria rapid assay |
| - *Phleboviruses* | Suckling mouse brain inoculation |
| - *Nairoviruses* | Viral culture (Vero cell line) |
| - *Paramyxoviruses* |  |
| - *Polyomaviruses* |  |
| - *Reoviruses* |  |
| - *Rhabdoviruses* |  |
